# Tailoring interventions to implement recommendations for the treatment of elderly patients with depression: a qualitative study

**DOI:** 10.1186/s13033-015-0027-5

**Published:** 2015-09-12

**Authors:** Eivind Aakhus, Ingeborg Granlund, Andrew D. Oxman, Signe A. Flottorp

**Affiliations:** Centre for Old Age Psychiatric Research, Innlandet Hospital Trust, 2312 Ottestad, Norway; Norwegian Knowledge Centre for the Health Services, Box 7004 St Olavs plass, 0130 Oslo, Norway; The Department of Health Management and Health Economics, University of Oslo, P.O Box 1089, Blindern, 0317 Oslo, Norway

**Keywords:** Primary health care, Depression, Elderly patients, Determinants of practice, Tailored implementation

## Abstract

**Background:**

To improve adherence to evidence-based recommendations, it is logical to identify determinants of practice and tailor interventions to address these. We have previously prioritised six recommendations to improve treatment of elderly patients with depression, and identified determinants of adherence to these recommendations. The aim of this article is to describe how we tailored interventions to address the determinants for the implementation of the recommendations.

**Methods:**

We drafted an intervention plan, based on the determinants we had identified in a previous study. We conducted six group interviews with representatives of health professionals (GPs and nurses), implementation researchers, quality improvement officers, professional and voluntary organisations and relatives of elderly patients with depression. We informed about the gap between evidence and practice for elderly patients with depression and presented the prioritised determinants that applied to each recommendation. Participants brainstormed individually and then in groups, suggesting interventions to address the determinants. We then presented evidence on the effectiveness of strategies for implementing depression guidelines. We asked the groups to prioritise the suggested interventions considering the perceived impact of determinants and of interventions, the research evidence underlying the interventions, feasibility and cost. We audiotaped and transcribed the interviews and applied a five step framework for our analysis. We created a logic model with links between the determinants, the interventions, and the targeted improvements in adherence.

**Results:**

Six groups with 29 individuals provided 379 suggestions for interventions. Most suggestions could be fit within the drafted plan, but the groups provided important amendments or additions. We sorted the interventions into six categories: resources for municipalities to develop a collaborative care plan, resources for health professionals, resources for patients and their relatives, outreach visits, educational and web-based tools. Some interventions addressed one determinant, while other interventions addressed several determinants.

**Conclusions:**

It was feasible and helpful to use group interviews and combine open and structured approaches to identify interventions that addressed prioritised determinants to adherence to the recommendations. This approach generated a large number of suggested interventions. We had to prioritise to tailor the interventions strategies.

## Background

Only 50 % of patients with depression receive care in accordance with guidelines [1, 2]. Many factors may impede or facilitate adherence and determine whether a patient receives appropriate care. These factors are referred to as determinants of practice [3]. Knowledge about determinants of practice can guide efforts to develop and choose interventions that are tailored to address those determinants and more effectively implement guidelines. Applying and increasing knowledge about effective strategies for implementing guidelines can potentially reduce the gap between scientific evidence and clinical practice.

The aim of the Tailored Implementation for Chronic Diseases (TICD) project was to directly compare alternative approaches in the tailoring process and subsequently assess the effectiveness of tailored implementation interventions [3]. The Norwegian part of TICD addressed elderly patients with depression [4]. Elderly patients with depression have an increased risk of a chronic course, and the prognosis is worse as compared with younger adults [5, 6]. Evidence indicates that healthcare professionals use longer time to diagnose depression and initiate adequate treatment in elderly patients [7].

Adherence to guidelines for depression improves patient outcomes [8, 9]. A logical step to improve adherence to guidelines is to identify significant determinants of practice and tailor implementation interventions to address these factors. Tailored interventions are more likely to improve professional practice than no intervention or dissemination of guidelines alone [10]. However, it is uncertain how best to tailor interventions. Thus, there is a need to compare different ways of identifying determinants and developing implementation strategies to address those determinants.

We conducted a systematic review of 13 clinical practice guidelines for the management of depression [11]. With the help of a reference group (see “Acknowledgments”), we prioritised six recommendations that we wanted to implement (Table 1). Depression in the elderly is frequent, affecting 10–16 % of people over 65 years, and complex, triggered by social, psychological, and biological factors [12, 13]. Acknowledging this complexity, the recommendations addressed the need for a coordinated combination of interventions, including pharmacotherapy, psychotherapy, self-help strategies, social strategies, and coordination of care. As a result of the prioritising process in a previous part of this project only treatment issues, and not diagnostic, were chosen [14]. Although the evidence for the effectiveness of antidepressants in depression has been questioned [15], there are systematic reviews that indicate that antidepressants are beneficial in combination with psychotherapy in elderly patients with severe depression [16, 17].

**Table 1:** Six prioritised recommendations for managing depression in the elderly in primary care

Prioritised recommendations	Full recommendation to be discussed in the groups and interviews
1. Social contact	Primary care physicians and other health care professionals should discuss social contact with elderly patients with depression, and recommend actions (e.g. group activities) for those who have limited social contact When needed, regular social contact with trained volunteers, recruited from centres for voluntary organisations, the red cross, mental health or community day care centres When possible, the patient’s relatives should be involved in the plan to improve social contact
2. Collaborative care plan	All municipalities^a^ should develop a plan for collaborative care for patients with moderate to severe depression. The plan should describe the responsibilities and communication between professionals who have contact with the patient, within primary care and between primary and specialist care. In addition, the plan should appoint depression case managers who have a responsibility for following the patient. The plan should describe routines for referral to specialist care
3. Depression case manager	Primary care physicians should offer patients with moderate to severe depression regular contact with a depression case manager
4. Counselling	Primary care physicians or qualified health care professionals should offer advice to elderly patients with depression regarding: Self-assisted programs, such as literature or web-based programs based on cognitive behavioural therapy principles Structured physical activity programmes, individually or group-based Healthy sleeping habits Anxiety coping strategies Problem solving therapy
5. Mild depression	Primary care physicians should usually not prescribe antidepressants to patients with mild depression. Primary care physicians may consider prescribing antidepressant medication to patients who suffer from a mild episode of depression and have previously responded to antidepressant medication when moderately or severely depressed
6. Severe depression, recurrent and chronic depression and dysthymia	Primary care physicians should offer these patients a combination of antidepressant medication and psychotherapy. If the physician is not trained to provide the patient with psychotherapy, patients should be referred to trained health care professionals

^a^Municipalities are the atomic unit of local government in Norway and are responsible for outpatient health care services, senior citizen services, and other social services. There are 429 municipalities

We identified 352 determinants of practice for the six recommendations using a multi-methods approach, and prioritised 99 determinants that we wanted to address by tailoring interventions to facilitate adherence to the recommendations [14]. The aim of this article is to describe how we developed implementation interventions based on these determinants of practice. We will evaluate the effectiveness of these interventions in a randomised trial [4], and conduct a process evaluation to examine the validity of the tailoring methods that were used [18]. Partners in the TICD project are conducting parallel studies addressing the implementation of guidelines for different chronic conditions in four other European countries. The sequential steps in the TICD project, the Norwegian publications focused on improving treatment of elderly patients with depression, and the cross-country TICD publications are summarised in Table 2.

**Table 2:** Sequential order of publications related to the various stages of the TICD project

Research	Norwegian (elderly patients with depression) publications	Cross-country TICD publications
Project protocol		Wensing et al. [3] (protocol)
Identification of determinants	Aakhus et al. [14]	Krause et al. [34]
Selection of interventions to address the identified determinants	This paper	Huntink et al. [20], Wensing et al. [35]
Cluster randomised trials to address the identified determinants	Aakhus et al. [4] (Study protocol) (a report of the results has not yet been completed or submitted for publication)	Baker et al. [10] (a future update of this review will include the results of the TICD trials)
Process evaluations to address the validity of the tailoring methods	A report of the process evaluation has not yet been completed or submitted for publication	Jager et al. [18] (protocol) (a report of the results has not yet been completed or submitted for publication)

## Methods

The TICD group developed a common protocol for the study. Each of the five countries in the TICD project selected between eight and 30 determinants for discussion in focus groups. The methods, setting, study sample and group interviews are presented in detail elsewhere [19, 20]. Here we briefly describe how we conducted our study in Norway.

We (EA, SF and AO) selected 22 of the 99 prioritised determinants of that we had identified in a previous study [14]. We used the tools provided by Flottorp and colleagues (TICD Worksheet 3: development of an implementation strategy) [21]. We independently assessed each of the 99 determinants by evaluating its likely impact (3 = major impact, 2 = moderate impact, 1 = minor impact) and the effect of the likely impact on adherence using a 7 point scale (−3 = major reduction in adherence, 0 = no effect in adherence and +3 = major increase in adherence) [21, 22]. This process yielded a product of the likely impact of the determinant and the likely effect on adherence (range −9 to +9). We then discussed potential implementation strategies, the likely impact of the implementation strategy, the feasibility of the implementation strategy and whether the strategy should be targeted (only implemented for selected GPs, practices or communities where the determinant could be identified) or adjusted. In addition, we assessed each determinant in light of what we could accomplish within the resources and the timeframe of the project. We also assessed what might be realistic based on our knowledge of the Norwegian primary healthcare system. Thus, the prioritised determinants were not a result of the scoring process alone. We resolved disagreement by discussion.

We developed a draft of a plan with 55 interventions that addressed all the 22 prioritised determinants and the six recommendations. We grouped the 55 interventions in the plan in six different categories (Box 1).

**Box 1:** Six domains in which interventions were put

Domain
1. Support for a collaborative care plan for elderly patients with moderate or severe depression
a. Development of the plan (offer templates and reminders that were essential for the plan, and that could be tailored to each municipality)
b. Content of the plan (suggested content, including recommendations, that describes the management of depression in the elderly that the municipality could include in the plan)
2. Resources for GPs and other health care personnel (leaflets, templates, manuals)
3. Resources for patients and their relatives (leaflets, manuals)
4. Outreach visits for GPs (presentation of recommendations, the evidence for the recommendations, determinants of practice for the recommendation and any local circumstances that may impede or facilitate adherence that would imply an adjustment of the strategy to local determinants)
5. Educational courses for GPs, other health care professionals, patients and their relatives, including CME courses for GPs and courses approved for nurses and other healthcare professionals
6. Online services (a web-site with all the resources, including e-learning courses)

**Fig. 1:**
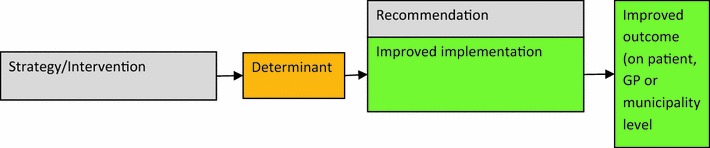
Logic model: general principle of the logic model

We grouped all interventions that the participants suggested into the six categories in the drafted plan. As described above, the intervention plan by the research team included 55 interventions that addressed all the prioritised determinants and could be fit within the six intervention categories [14]. This is presented in the logic model (Fig. 1; “Appendix”).

The logic model is a construct that connects the planned interventions to the determinants and the assumed effects of the interventions. One or more interventions may address one or more determinants.

We presented the selected 22 determinants to the groups (Table 3). We did not present our draft plan to the participants, to avoid influencing their thinking.

**Table 3:** Prioritised determinants to six recommendations presented to the focus groups

Recommendation	Determinant [14]
Social contact	Finding volunteers
	Lack of awareness of local community/services
	Social withdrawal in elderly patients with depression
	Lack of connection between the patient and volunteers
	Requires organising the service
Collaborative care plan	1. Actionable plans with shared ownership increases the plan’s feasibility
	2. Lack of coordination within municipalities, especially between GPs and other municipal services
	3. Implementation of the plan
Depression case manager	1. A description for how the doctor should proceed
	2. Good relationship between patient and depression case manager
	3. If the person is completely alone in the task
Counselling	1. GP’s time constraint
	2. Health professionals believe self-help program is not beneficiary for this population
	3. There is a shortage of this type of service
	4. Lack of expertise for counselling among GPs and other health professionals
Antidepressants in mild depression	1. GPs time constraint
	2. Patient information that drugs do not help in mild depression
	3. Difficult to reverse a trend where the doctor has been told that they prescribe antidepressants too seldom
	4. Lack of other types of services makes it difficult to adhere
	5. GP wants to “do something”, drugs are simple actions
Severe, recurrent and chronic depression, dysthymia	1. GPs do not have this expertise (psychotherapy)
	2. Elderly are not prioritised for this type of service
	3. Lack of health professionals who can provide this type of service

### Setting and sample

We conducted six group interviews, one for each of the following stakeholder groups: general practitioners (GPs), primary health care nurses, implementation researchers, quality improvement officers, professional and voluntary organisations, and relatives of elderly patients with a present or past history of depression. In an earlier part of the project we experienced that elderly patients with present or past depression found the interview questions difficult and we terminated recruitment of patients earlier than planned [14]. Based on this experience, for this study we invited relatives of patients rather than patients. We contacted 17 individuals, 12 research or health care organisations, and eight stakeholder organisations by phone and subsequently email if they asked for further information. We invited people to participate or to suggest a colleague or a representative. After the groups sessions we asked each participant to rate whether they found their participation meaningful, useful and satisfactory.

### Group interviews

We sent written material with the recommendations and information about how the groups would be organised to the participants in advance, and asked them to prepare for the group interviews. The group interviews followed a standardised procedure according to the common TICD protocol, although the content of the questions and responses differed across countries [19]. The interviews lasted 120 min and consisted of a brainstorming session followed by a structured interview phase. EA facilitated the groups. SF or IG were co-leaders, recorded all items, made field notes and asked questions as prompts when needed. First, EA introduced the project, the recommendations and information on the performance gap between clinical practice and the recommendations. Then each participant received a sheet with the six recommendations and the prioritised determinants. The participants brainstormed individually for 10 min and wrote down ideas for interventions to address each determinant for each recommendation. The group members then presented their suggested interventions to the group. EA recorded the suggestions for each determinant on a whiteboard. Following the principles of brainstorming we tried to avoid criticism, while we encouraged combining and extending previously suggested items [23]. There was no limit to the number or type of the items. After a short break, EA briefly presented current knowledge regarding effectiveness of strategies to implement depression guidelines. We then conducted the structured part of the focus group. EA instructed the participants to discuss the interventions that they had suggested through the brainstorming session, to add others, and to prioritise the suggested interventions.

We based the prioritisation process on the following considerations:

1. Perceived importance of the targeted determinant
2. Perceived impact of the implementation intervention
3. Research evidence underlying the effect of the intervention
4. Feasibility and cost of the intervention
5. Other considerations

### Deviation from the common TICD protocol

We tried to conduct the first group interview in accordance with the common protocol for the five national TICD projects. Following the first group interview, we realised that in our project it was not feasible to address each of the suggested interventions in the systematic way stated in the protocol due to the large number of suggested determinants and interventions, and a limited amount of time. We chose a more pragmatic approach for the remaining group interviews. We asked the groups to prioritise the interventions that we had recorded on the whiteboard, bearing in mind the considerations mentioned above throughout the procedure. The first four groups (implementation researchers, nurses, quality improvement officers, and representatives of organisations) followed the same sequence, starting with the first recommendation (social contact) and the first determinant (finding volunteers) (Table 1).

We observed that the number of suggested interventions decreased as the session moved on. The groups produced fewer ideas for mild and severe depression (recommendations number five and six) as compared with social contact (the first recommendation). Thus, we asked the fifth group (relatives) to focus mainly on the first three recommendations (social contact, collaborative care plan, depression case manager) because we believed that these recommendations were most relevant to this group. We asked the last group (GPs) to focus on the last three recommendations (counselling, mild depression and severe depression), starting with the last determinant for the last recommendation.

### Analysis

We applied a five-step framework for our analysis [24]:

1. *Familiarisation* We audiotaped all group sessions, photographed the results from the whiteboard, and made short notes from the discussions. We used the whiteboard results as the primary source of information for the analysis. We used the audio-recordings and the notes to include additional material that we had not recorded on the board. EA put all quotes containing suggested interventions in tables, one column for each group and one table for each recommendation. SF and EA reviewed these Tables 
2. *Identifying a thematic framework* We used the drafted plan for an intervention package (Table 4, left column) as a comparator.

**Table 4:** A complex intervention plan developed by the research team and the modifications and new interventions suggested by the focus groups for each recommendation

Recommendation: social contact			
Draft plan from the research team	Modifications or new interventions from the group sessions	Adaptation to municipalities or practices	Targeted determinant (see Table 3)
*Collaborative care plan* ^a^. Include key personnel, e.g. leaders for voluntary organisations who can help identifying voluntary	Such as Centre for volunteers, Centre for healthy life, charity organisations (Lions, Red Cross), congregations and fitness centres	Identify key personnel in each municipality	Finding volunteers
*Collaborative care plan*. Provide a model agreement between the municipality and voluntary organisations that clarifies expectations, responsibilities follow-up and monitoring	Provide a model agreement between the municipality and voluntary organisations that clarifies expectations, responsibilities (such as a contact or an office), communications (such as, for instance, a website, neighbourhood/local newspaper, posters), follow-up and monitoring		
*Educational resources*. Educate voluntary in communication with depressed patients			
	*Collaborative care plan.* Inform relatives, use existing local knowledge within the community (e.g. home-based nurse staff, voluntary organisations, congregations)	Identify persons who possess local knowledge on voluntary organisations and volunteers	
*Collaborative care plan*. Include key personnel	Such as family, GP, home based nursing services, health centre for the elderly, municipality’s cultural agency, Council or the elderly and the union for retirees.		Lack of awareness of local community/services
*Collaborative care plan*. Help to obtain an overview of services in the community	Such as obtain an overview in one place, e.g. by the home based nurse services administration, responsible for contacting voluntary organisations for an overview		
	*Collaborative care plan.* Provide information via brochures, advertisements in the local newspaper, the municipalities’ website	Information tailored^b^ to each community	
	*Collaborative care plan.* Outreach activities (e.g. letter to all over 80, information in the media		Social withdrawal in elderly patients with depression
*Resources for patients and their relatives.* Information to patients and their relatives on social contact, alternatives to antidepressants and counselling	Such as brochures aimed at patients and their families, contacting elderly who do not attend consultations or their relatives)		
	*Collaborative care plan.* Describe the role of senior centres and health clinics for the elderly to reduce social withdrawal		
*Resources for GPs and other health care professionals*. Provide contact information for physical activity, voluntary organizations, senior centres, etc.	e.g. a contact/coordinator of the municipal/district, using brochures	Templates for how the municipality could publish contact	Lack of connection between the patient and the volunteer
*Collaborative care plan.* Create a job description that helps the municipality to find suitable persons who can lead the efforts		Create templates with a job description that each municipality could fit to local routines	Requires organisation
*Collaborative care plan.* Consider the financial resources to motivate people to take this work			

Recommendation: collaborative care plan			
Draft plan from the research team	Modifications or new interventions from the group sessions	Adaptation to municipalities or practices	Targeted determinant (see Table 2)
*Collaborative care plan.* Including key personnel in the development of the plan	Key personnel such as coordinator/office for approval of health services, GP/GP committees, Community based psychiatric centres, and impose key personnel to help in the development of the plan	Template for the plan should be adapted to each municipality and include key personnel	Actionable plans with shared ownership increases the plan’s feasibility
*Collaborative care plan.* Include The Norwegian Association of Local and Regional Authorities (KS) and local opinion leaders in the work with the plan and presentation of recommendations			
*Collaborative care plan.* Help to make it convenient to implement the plan (e.g., to create a comprehensive plan for psychiatry, where seniors also have a place)			
*Collaborative care plan*. Help to develop a dissemination and implementation plan			
	*Collaborative care plan*. The plan must be consistent with the national collaboration reform		
*Collaborative care plan.* Exchange experiences (good/bad) across municipalities			
*Online services*. Support for electronic communication between health care personnel in the community and specialists if possible			Lack of coordination within municipalities, especially between GPs and other municipal services
*Collaborative care plan.* Help to develop a dissemination and implementation plan			Implementation of the plan
*Collaborative care plan—content.* Describe the recruitment of care managers to obtain suitable personnel (use local knowledge to identify particularly suitable people)		Provide templates for a job description that could be adapted to each municipality and provide help to identify suitable professionals	
*Collaborative care plan—content.* Clarify the individual tasks with clear guidelines and support for them to adhere	Assign one person to the responsibility for the plan (e.g. CMO)	Name the person or the applied role in the system that carry the responsibility for the plan	
*Collaborative care plan—development.* Include The Norwegian Association of Local and Regional Authorities (KS) and local opinion leaders in the work with the plan and presentation of the recommendations	The plan should be politically/administratively anchored		
*Collaborative care plan*—*content.* Help to implement the plan in practice	e.g. through regular meetings. If necessary; compel health professionals to implement the plan		
*Collaborative care plan.* Arrangements for monitoring and evaluation of the plan (e.g. via notification systems, involving health committee)			
*Collaborative care plan—development.* A model plan with a checklist of both the process to make the plan and the content of the plan			
*Online services.* Web page with all the resources and recommendations			
*Collaborative care plan.* Arrangements for dissemination and implementation of the plan			

Recommendation: depression case manager			
Draft plan from the research team	Modifications or new interventions from the group sessions	Adaptation to municipalities or practices	Targeted determinant (see Table 2)
*Outreach visits to GPs.* Inform GPs about the concept and evidence supporting the CM, and how referral should be done			A description for how the GP should proceed
*Resources for GPs and other health care professionals*—Structured referral forms to case manager, web-based and integrated in journal		Provide templates for referral that can be adjusted^b^ to each municipality	
*Collaborative care plan*—*content*. Establish CM services in each municipality and effective referral practices of GPs to CM	Consider initiating contact between doctor, patient and CM. CM can be a GP assistant in the GP practice or another appropriate person in primary care		
*Collaborative care plan*—content. A plan for support/guidance/counselling for CM			Good relationship between patient and depression case manager
*Educational resources.* Training in communication with depressed patients for CMs			
	*Educational resources.* Inform CM that family members should be involved when necessary		
*Collaborative care plan*—content. A plan for support/guidance/counselling for CM	e.g. establish groups for CMs, supervised by GPs, psychiatric nurses or specialist health care		If the person is completely alone on the task
*Online services*. Integrate recommendations and resources to medical records systems			

Recommendation: counselling			
Draft plan from the research team	Modifications or new interventions from the group sessions	Adaptation to municipalities or practices	Targeted determinant (see Table 2)
*Outreach visits to GPs*. Discuss physician time constraints and the possibility of extended consultations and additional fees			GPs’ time constraint
*Outreach visits to GPs.* Clarify to GPs that older with moderate to severe depression profit from counselling		Target^b^ and adjust this information to each outreach visit	
	*Outreach visits to GPs.* Consider if other health professionals than GPs can offer counselling	Identify personnel that exhibit these skills in each municipality during outreach visits	
*Outreach visits to GPs.* Emphasize for GPs that we have alternatives to antidepressants for mild depression that are more effective and less harmful			Health professionals believe self-help program is not beneficiary for this population
*Resources for general practitioners and other health care professionals.* Resources for counselling (e.g. brief information about self-help programs, physical activity, sleep habits and anxiety coping that can be discussed with patients and caregivers, use simple forms or manuals	*Resources for general practitioners and other health care professionals.* Resources for counselling (e.g. brief information about self-help programs, physical activity, sleep habits and anxiety coping that can be discussed with patients and caregivers, use simple forms or manuals		There is a shortage of this type of service
	Collaborative care plan. Identify services to determine if it is right that the services are missing	As part of the plan	
	*Outreach visits.* Identify services to determine if it is right that the services are missing	As part of outreach visits	
*Resources for general practitioners and other health care professionals.* Resources for counselling (e.g. brief information about self-help programs, physical activity, sleep habits and anxiety coping that can be discussed with patients and caregivers, use simple forms or manuals			Lack of skills to provide counselling among GPs and healthcare
*Educational resources.* Courses for GPs must merit for the speciality (CME credits) (15 h) and can be a combination of web-based courses and educational meetings			
*Educational resources.* E-learning courses and other forms of informing healthcare professionals about the recommendations and in particular techniques for counselling and motivation,	Training for GPs should be designed as a clinical topic course and merit for CME credits		

Recommendation: antidepressants in mild depression			
Draft plan from the research team	Modifications or new interventions from the group sessions	Adaptation to municipalities or practices	Targeted determinant (see Table 2)
*Outreach visits to GPs*. Discuss physician time constraints and the possibility of extended consultations and additional fees			GPs’ time constraint
*Resources for patients and their relatives.* Information to patients and their relatives on social contact, alternatives to antidepressants and counselling	e.g. information presented in brochures and on websites	Information forms that allow the GP to tailor information to patients	Patient information that drugs do not help in mild depression
*Outreach visits to GPs.* Provide evidence for not using antidepressants for mild depression and inform that we have better alternatives			Difficult to reverse a trend where the doctor has been told that they prescribe antidepressants too rarely
*Outreach visits to GPs.* Emphasize for GPs the need for grading the severity of depression using appropriate tools, such as MADRS, for diagnosis and follow-up			
*Outreach visits to GPs.* Discuss the idea that GPs feel that they are accused of prescribing antidepressants too seldom			
	Resources for GPs and other healthcare professionals. Offer monitoring and feedback to GPs, preferably in groups	Use existent groups or discuss with leaders of local GP groups whether new groups could be created	
*Educational courses.* Provide training in counselling as problem solving therapy, anxiety coping and sleep habits, for instance as e-learning courses			Lack of other types of services makes it difficult to adhere
*Educational courses.* Courses for GPs must merit for the speciality (15 h) and can be a combination of web-based courses and meetings			
*Educational courses.* E-courses and other courses to inform healthcare professionals about the recommendation, and in particular techniques for counselling and motivation			
*Outreach visits to GPs.* Discuss this with GPs. Suggest strategies to avoid prescribing antidepressants			GP wants to “do something”, drugs are simple actions

Recommendation: Antidepressants and psychotherapy in severe and recurrent depression			
Draft plan from the research team	Modifications or new interventions from the group sessions	Adaptation to municipalities or practices	Targeted determinant (see Table 2)
*Resources for general practitioners and other health care professionals.* Structured referral forms to psychotherapy	to private specialists, district based psychiatric centres and old age psychiatry	Templates for referral may be adjusted to each municipality	GPs do not have this expertise (psychotherapy)
*Resources for patients and their relatives.* Information to patients and their families about the combined treatment (psychotherapy and antidepressants)			Elderly are not prioritised for this type of service
Collaborative care plan—development. Include key personnel in the development of the plan (managers, administrators, specialists in private practices, GPs, GPs’ committees, nurses, specialist care, patients and relatives)			
*Collaborative care plan—content.* A clear message in the plan about access to psychotherapy for the elderly with severe depression with community based psychiatric centres and private practitioners		Templates for the description of specialist care adjusted to the municipality and the collaborating specialists/specialist services	
*Collaborative care plan—content.* State that the recommendations are in accordance with national guidelines	e.g. in the media		
Outreach visits. Clarify that older with moderate to severe depression profit from psychotherapy			
Educational courses. Training in cognitive therapy for general practitioners and psychiatric nurses for those who want it			Lack of health professionals who can provide this type of service
Resources for GPs and other healthcare professionals. Structured referral forms to psychotherapy		Templates for referral forms adjusted to each municipality	

^a^For a comprehensive description of the various items in the intervention plan, please refer to the methods section

^b^In this table we use the terms “tailoring”, “targeting” and “adjustment”. We define these terms in the following way: Tailoring: planning interventions/strategies that are designed to achieve desired changes in healthcare practice based on an assessment of determinants of healthcare practice. Targeting: implementation of the tailored intervention for selected GPs, practices or communities (where the determinant could be identified) and not for others (where the determinant could not be identified). Adjustment: modification of the tailored intervention to address determinants that are identified as the tailored intervention is implemented
3. *Indexing* SF and EA independently analysed these data, assessing whether the interventions that we identified during each session were similar or different from each other or the intervention plan drafted by the research team. We categorised the interventions using the format of the drafted plan.
4. *Charting* We discussed our assessments and revised the final list of interventions for each determinant based on a consensus. EA linked the suggested interventions to the drafted plan, either as modifications of interventions already described or as new suggestions.
5. *Mapping and interpretation* We all reviewed the revised intervention plan and grouped the interventions across recommendations and the TICD checklist items in order to identify any topics of related suggestions in the data-set [21]. We used a standardised procedure to rank the interventions according to the following criteria:
  1. Is it feasible? (Score 1 = Yes, 2 = Maybe, 3 = No)
  2. Will it help? (Score 1 = Yes, 2 = Maybe, 3 = No)

This yielded a score for each intervention (range 2–6). A lower score indicated that a suggested intervention was both feasible and helpful. Finally, we assessed the score for each intervention and asked whether we should prioritise the intervention for the planned trial (Yes/No) and, if so, if we should adjust it to each municipality or practice (Yes/No)? We resolved disagreement by discussion.

## Results

Thirty-one people consented to participate. Two did not show up (one sick, the other gave no reason). Thus, 29 people participated in the various group sessions (five GPs, four implementation researchers, six primary care nurses, six representatives from professional and voluntary organisations, five quality improvement officers and three relatives of elderly patients). Making personal calls was a practical and effective strategy to recruit participants. Three people living in other parts of Norway participated in the group interviews using Skype. The group sessions took place from October to December 2013. The groups were enthusiastic, relaxed and creative. Written feedback from 23 (80 %) of the participants indicated that they experienced the group sessions as meaningful. The limited time was a challenge and several claimed that they should have prepared better prior to the meeting. One Skype participant found it difficult to contribute to the structured part of the session for technical reasons. A majority (70 %) said that their participation was useful and 68 % said it was satisfactory. None indicated that their participation was unsatisfactory or not useful.

The six groups yielded approximately 450 suggested interventions, of which many were related to each other and to suggested interventions in the drafted plan. We found that approximately 70 suggestions contained statements or attitudes rather than interventions (such as “Lack of available services is more important than GPs’ time constraints”.). This left 379 suggestions of interventions for further analysis. In the first four group interviews, we presented the recommendations in the following order: social contact, collaborative care plan, depression case manager, counselling, mild depression and severe depression. There were 127 suggestions for interventions to improve adherence to the recommendation on social contact, 68 for collaborative care plan, 54 for depression case manager, 40 for counselling, 47 for mild depression, and 43 for severe depression. The groups with representatives of professional and voluntary organisations and quality improvement officers generated the most suggestions, (106 and 96 respectively). The group with nurses generated 67 suggestions and the implementation researchers 36. The groups with GPs and relatives focused mainly on three selected recommendations. They generated 41 and 33 suggestions respectively.

We reduced the number of interventions from 379 to 65 based on our assessments of their likely effectiveness and feasibility. Of these, 28 were added or modified after the group sessions (18 modifications and 10 new interventions). We determined that 18 of the interventions should be adapted to municipalities or practices (Table 4). In Table 4, we present the following for each recommendation: the research group’s suggestions for interventions prior to the group interviews (first column), modifications of these suggestions and new suggestions from the group interviews (second column), whether the intervention should be adapted to each municipality or practice and, if so, how (third column), and the determinant at which each intervention was targeted (last column).

We removed suggestions that could not be tested in the planned randomised controlled design [4], such as “dissemination in the Norwegian Electronic Medical Handbook” and “using newspapers to inform patients”. Some suggestions were related, although addressing different recommendations and determinants. We were able to group several interventions together. We reduced the number of interventions presented in the logic model from 65 (the sum of interventions suggested by the research team and the groups) to 52 by combining all similar or related interventions that addressed different determinants or recommendations as presented in the logic model (“Appendix”). Each intervention is numbered in the order it appears the first time. When a closely related intervention is noted later in relation to a different recommendation or determinant, it is given the same number and a sequential lowercase letter. Thus intervention 1a is closely related to 1b, 1c and 1d, although they may not appear in numerical order. An intervention could address a single determinant, such as this suggestion: “Discuss GPs’ urge to do something and the view that prescribing antidepressants is a simple action in outreach visits”, which addressed this determinant: “GPs want to do something, and prescribing drugs is easy”. An intervention could also address several determinants, such as: “Provide structured referral forms for psychotherapy”, which addressed these two determinants: “GPs do not have the expertise to provide psychotherapy” and “There is a lack of health professionals who can provide this type of service”.

Several interventions could also address the same determinant. For example, this determinant: “There is a lack of other types of services that makes it difficult not to prescribe antidepressants in mild depression” was addressed by these three interventions: “Provide training in counselling to health professionals”, “Provide CME approved courses in counselling to GPs”, and “Provide courses as e-learning courses”.

## Discussion

We have conducted group interviews with several stakeholder groups to inform our decisions about how to tailor implementation interventions to improve adherence to clinical practice guidelines for elderly patients with depression. We developed a draft plan consisting of 55 interventions that addressed determinants of practice for the six recommendations, organised in six domains: resources for the development of a collaborative care plan, resources for GPs and other healthcare professionals, resources for patients and their relatives, outreach visits to GPs, educational resources for GPs and web-based services. The plan covered many of the interventions that the groups suggested. However, the groups added many new ideas, and they modified approximately half of the interventions suggested in the draft plan.

## Strengths and limitations

We included several stakeholder groups, to achieve a purposeful sample of healthcare professionals, relatives of elderly patients with depression, implementation researchers and others that might be able to suggest effective interventions to address the identified determinants of practice. This approach to tailoring an intervention to prioritised determinants was standardised across five countries and disease groups in the TICD project [4, 25–28]. We are not aware of any other project that has addressed tailoring of implementation interventions in this comprehensive manner, using a check-list systematically to identify and prioritise determinants of practice and to identify interventions that could address them [21]. However, due to the complexity of our recommendations, the large number of prioritised determinants and the limited time available for the interviews, it was not feasible for us to address each of the suggested interventions in the systematic way stated in the common TICD protocol.

The recommendations that we prioritised addressed several levels of the healthcare system, from the patients and their relatives to the healthcare professionals and the healthcare administration in the municipalities. The use of the TICD checklist to prioritise determinants and interventions made it possible to analyse the results in a systematic way [21]. Nonetheless, the results from this part of the analysis were assessments based on our considerations and judgments. An alternative strategy would be for representatives from the stakeholder groups to do this assessment. The wide range and the large number of interventions that the groups discussed within a limited time may have compromised more detailed and structured discussions, and may have resulted in superficial assessments for some determinants or interventions.

The number of suggested interventions for each determinant and recommendation varied. Recommendations presented early in the session appeared to yield the most suggestions. These recommendations addressed mainly the community and municipalities, while the last recommendations addressed clinicians. It is possible that the nature of the first guidelines generated more suggestions. It is also possible that there were fewer suggestions for the recommendations presented later in the interview because of exhaustion in the groups. One solution to this could have been to present the recommendations in a different order for each of the groups.

The large number of suggested interventions addressed only six recommendations, whereas clinical practice guidelines frequently contain many more recommendations. There is a risk that guideline developers will experience information overload, if they try to use this approach for a full guideline.

We excluded suggested interventions that could not be evaluated in our planned cluster randomised controlled trial. Thus, we omitted potentially useful dissemination channels suggested by the groups, such as media and e-resources that are popular among healthcare professionals (The Norwegian electronic health library, the Norwegian Directorate of Health’s web site and the Norwegian Electronic Medical Handbook).

## Comparison with existing literature

Determinants of practice related to depression guidelines are numerous and apply to all levels of the healthcare system [14, 29, 30]. Relatively few studies on improving the care of patients with depression have described the development of a systematically planned intervention tailored to address identified determinants. Shirazi and colleagues [31] demonstrated that tailoring an educational intervention, based on GPs’ readiness-to-change (high-low), improved GPs’ performances in hypothetical (role-playing) consultations as compared with controls. Verhaak and colleagues [32] found that disability (particularly disability that affects participation, self-care and social activities) had a major impact on depression in the elderly. One might argue that the interventions that we planned considered this aspect to a limited degree only. Nevertheless, we addressed social withdrawal and frailty in our planned interventions. Furthermore, their findings indicated that the effect of disability on depression was largest among the younger elderly (those between 60 and 70 years). We included patients 65 years or older in our study. In a randomised controlled trial based on a psychological theoretical framework, Baker and colleagues [33], identified obstacles to adherence among 34 GPs, and tailored their intervention to each practitioner. They found that this strategy improved assessment of suicide risk and depression, assessed with Beck’s Depression Inventory. They found no difference for anti-depressant therapy or utilisation of psychotherapeutic services. Addressing clinicians individually to identify determinants of practice is an attractive approach, but rarely realistic in large-scale efforts to implement clinical practice guidelines. We deemed this approach unfeasible.

In a joint analysis of the studies to tailor interventions in the TICD project, Huntink and colleagues [20] found no relationship between the total number of suggested interventions and the number of unique suggestions (interventions only suggested by one group).

## Implications for research and practice

The extent to which there are similar determinants of practice in other settings and the extent to which similar interventions would be appropriate in other settings is uncertain. However, many of the same determinants are likely to be similar in other settings. The approach that we used to develop a package of tailored implementation interventions was both feasible and efficient. Those interested in tailoring interventions to implement guidelines can use the TICD checklist [21] and the interview methods that we used in this study.

We are evaluating the effectiveness of the tailored interventions that we have developed in a randomised trial [4]. We will assess whether we identified the most important determinants and selected appropriate interventions to address those in a process evaluation [18].

There is a paucity of research comparing different methods of identifying determinants of practice and tailoring implementation interventions to address those. To our knowledge, the TICD project is one of the first projects that have done this [19, 20, 34]. We need more research to improve the methods used to tailor interventions and to understand how best to do this. This includes evaluation of strategies to prioritise suggested interventions, given the abundance of suggestions that is possible, as illustrated by this study.

## Appendix

### Logic model

*Logic model, part A* General principle of the logic model and overview [4].

*Logic model, part B* The model (see explanation of the use of lowercase letters at the end of the figures).

*Explanation* Each intervention is numbered in the order it appears the first time. When a closely related intervention is noted later in relation to a different recommendation or determinant, it is given the same number and a sequential lowercase letter. Thus intervention 1a is closely related to 1b, 1c and 1d. The interventions are described in more detail in part C.* CCP-C* collaborative care plan—content,* CCP-D* collaborative care plan—development,* DS* data systems,* ER* educational resources,* OV* outreach visits,* RGP* resources for general practitioners and other healthcare professionals,* RPR* resources for patients and their relatives.

*Logic model, part C* Description of interventions

This table comprises a comprehensive description of each intervention. The numbers refer to the numbers in the figures. Closely related strategies are given identical numbers, with ascending lower case letters.

**Table:**

1a. *Collaborative care plan—development.* Include key personnel, e.g. leaders for voluntary organisations who can help identifying volunteers	2. *Collaborative care plan—development*. Provide a model agreement between the municipality and voluntary organisations that clarifies expectations, responsibilities (such as a contact or an office), communications (such as, for instance, a website, neighbourhood/local newspaper, “result”), follow-up and monitoring	3. *Educational resources.* Educate voluntaries in communication with depressed patients	4. *Resources for patients and their relatives.* Inform relatives, use existing local knowledge within the community (e.g. homebased nurse staff, voluntary organisations, *congregations*)	1b. *Collaborative care plan—development*. Include key personnel (e.g. families, GPs, home based nursing services, health centre for the elderly, municipality’s cultural agency, council for the elderly and retired)
5. *Collaborative care plan—development*. Help to obtain an overview of services in the community (collective overview in one place, e.g. by the home based nursing services administration, responsible for contacting voluntary organisations for an overview)	6a. *Resources for patients and their relatives.* Provide information e.g.via the council website, brochures and advertisements in the local newspaper	7. *Outreach visits.* Creative/alternative solutions for social contact (eg involving families, home care can identify depression)	8. *Resources for patients and their relatives.* Outreach activities (e.g. letter to all over 80)	6b. *Resources for patients and their relatives.* Information to patients and their relatives on social contact, alternatives to antidepressants and counselling (e.g. in brochures aimed at patients and their families, by contacting elderly who do not attend consultations or their relatives)
9. *Collaborative care plan—content.* Describe the role of senior centres and health clinics for the elderly in reducing social withdrawal	10. *Resources for general practitioners and other health care professionals.* Contact information for physical activity, voluntary organizations, senior centres, etc. (e.g. contact/coordinator of the municipal/district, using brochures)	11. *Collaborative care plan—development.* Create a job description that helps the municipality to find suitable persons who can lead the efforts	*12. Collaborative care plan—development.* Consider the financial resources to motivate people to take this work	*1c. Collaborative care plan—development.* Including key personnel in the development of the plan (e.g. coordinator/office for approval of health services, GP/GP committees, Community based psychiatric centres) impose key personnel to help in the development of the plan
13. *Collaborative care plan—development.* Include The Norwegian Association of Local and Regional Authorities (KS) and local opinion leaders in the work with the plan and presentation of recommendations	14*. Collaborative care plan—development.* Help to make it convenient to implement the plan (e.g., to create a comprehensive plan for psychiatry, where seniors also have a place	15. *Collaborative care plan—development.* Exchange experiences (good / bad) across municipalities	16a. *Collaborative care plan—development.* Help to develop a dissemination and implementation plan	17. *Collaborative care plan—content.* The plan must be consistent with the national collaboration reform
18. *Data systems.* Support for electronic communication between health care personnel in the community and specialists if possible	16b. *Collaborative care plan—development.* Help to develop a dissemination and implementation plan	19. *Collaborative care plan—content.* Describe the recruitment of care managers to obtain suitable personnel (use local knowledge to identify particularly suitable people)	20. *Collaborative care plan—content.* Clarify the individual tasks with clear guidelines and support for them to adhere, one person responsible for the plan (e.g. CMO)	21. *Collaborative care plan—development.* Include The Norwegian Association of Local and Regional Authorities (KS) and local opinion leaders in the work with the plan and presentation of the recommendations. The plan should be politically/administratively anchored
22. *Collaborative care plan—content.* Help to implement the plan in practice, e.g. through regular meetings. If necessary to compel health professionals to implement the plan	23. *Collaborative care plan—content.* Arrangements for monitoring and evaluation of the plan (e.g. via notification systems, involving health committee)	24. *Collaborative care plan—development.* A model plan with a checklist of both the process to make the plan and the content of the plan	25. *Data systems.* Web page with all the resources and recommendations	26. *Collaborative care plan—development.* Arrangements for dissemination and implementation of the plan
27. *Outreach visits to GPs.* Inform GPs about the concept and evidence supporting the CM, and how referral should be done	28a. *Resources for general practitioners and other health care professionals.* Structured referral forms to case manager, web-based	29. *Collaborative care plan—*content. Establish CM services in each municipality and effective referral practices of GPs to CM. Consider initiating contact between doctor, patient and CM. CM can be a GP assistant in the GP practice or another appropriate person in primary care	30a. *Collaborative care plan—*content. A plan for support/guidance/counselling for CM	31. *Educational resources.* Training in communication with depressed patients for CMs
32. *Educational resources.* Inform CM that family members should be involved when necessary	30b. *Collaborative care plan—*content. A plan for support/guidance/counselling for CMs (e.g. establishing supervision groups for CMs led by GPs, psychiatric nurses or specialist care)	33. *Outreach visits to GPs*. Discuss physician time constraints and the possibility of extended consultations and additional fees	34. *Outreach visits to GPs.* Clarify to GPs that older with moderate to severe depression profit from counselling	35. *Outreach visits to GPs.* Consider if other health professionals than GPs can offer counselling
36. *Outreach visits to GPs.* Emphasize for GPs that we have alternatives to antidepressants for mild depression that are more effective and less harmful	37a. *Resources for general practitioners and other health care professionals.* Resources for counselling (e.g. brief information about self-help programs, physical activity, sleep habits and anxiety coping that can be discussed with patients and caregivers, use simple forms or manuals	38. *Collaborative care plan—*content. Identify available services for the patients in the municipality to determine if it is right that the services are missing	37b. *Resources for general practitioners and other health care professionals.* Resources for counselling: brief info-material on self-help programs, physical activity, sleep habits and anxiety coping that can be discussed with the patient and their relatives/caregivers	37c*. Resources for general practitioners and other health care professionals.* Resources for counselling: simple forms/checklists
39. *Educational resources.* Training in counselling as PST, anxiety, coping and sleep habits, such as e-learning courses	40. *Educational resources.* Courses for GPs must merit for the speciality (CME credits) (15 h) and can be a combination of web-based courses and educational meetings	41. *Educational resources.* E-learning courses and other forms of informing healthcare professionals about the recommendations and in particular techniques for counselling and motivation, training for GPs should be designed as a clinical topic course (CME credits)	6c. *Resources for patients and their relatives.* Information to patients and their relatives on social contact, alternatives to antidepressants and counselling (e.g. written info in brochures, websites	42. *Outreach visits to GPs.* Provide evidence for not using antidepressants for mild depression and inform that we have better alternatives
43. *Outreach visits to GPs.* Emphasize for GPs the need for grading the severity of depression using appropriate tools, such as MADRS, for diagnosis and follow-up	44. *Outreach visits to GPs.* Discuss the idea that GPs feel that they are accused of prescribing antidepressants too seldom	45. *Educational courses.* Provide training in counselling as problem solving therapy, anxiety coping and sleep habits, for instance as e-learning courses	46. *Educational courses.* Courses for GPs must merit for the speciality (15 h) and can be a combination of web-based courses and meetings	47. *Educational courses.* E-learning courses and other courses to inform healthcare professionals about the recommendations and special techniques of counselling and motivation
48. *Outreach visits to GPs.* Discuss this with GPs. Suggest strategies to avoid prescribing antidepressants	49. *Educational courses.* Training in cognitive therapy for general practitioners and psychiatric nurses for those who want it	28b. *Resources for general practitioners and other health care professionals.* Structured referral forms to psychotherapy (to private specialists and Community based psychiatric centres and old age psychiatry	6d. *Resources for patients and their relatives.* Information to patients and their families about the combined treatment (psychotherapy and antidepressants)	1d. *Collaborative care plan—development.* Include key personnel in the development of the plan (managers, administrators, specialists in private practices, GPs, GPs’ committees, nurses, specialist care, patients and relatives)
50. *Collaborative care plan—content.* A clear message in the plan about access to psychotherapy for the elderly with severe depression with community based psychiatric centres and private practitioners	51. *Collaborative care plan—content.* A system for monitoring and evaluation of the plan	52. *Collaborative care plan—content.* State that the recommendations are in accordance with national guidelines		

## References

[1] Smolders M, Laurant M, Verhaak P, Prins M, van Marwijk H, Penninx B (2009). Adherence to evidence-based guidelines for depression and anxiety disorders is associated with recording of the diagnosis. Gen Hosp Psychiatry.

[2] Duhoux A, Fournier L, Nguyen CT, Roberge P, Beveridge R (2009). Guideline concordance of treatment for depressive disorders in Canada. Soc Psychiatry Psychiatr Epidemiol.

[3] Wensing M, Oxman A, Baker R, Godycki-Cwirko M, Flottorp S, Szecsenyi J (2011). Tailored implementation for chronic diseases (TICD): a project protocol. Implement Sci..

[4] Aakhus E, Granlund I, Odgaard-Jensen J, Wensing M, Oxman AD, Flottorp SA (2014). Tailored interventions to implement recommendations for elderly patients with depression in primary care: a study protocol for a pragmatic cluster randomised controlled trial. Trials..

[5] Adamson JA, Price GM, Breeze E, Bulpitt CJ, Fletcher AE (2005). Are older people dying of depression? Findings from the Medical Research Council trial of the assessment and management of older people in the community. J Am Geriatr Soc.

[6] Mitchell AJ, Subramaniam H (2005). Prognosis of depression in old age compared to middle age: a systematic review of comparative studies. Am J Psychiatry.

[7] Arean PA, Alvidrez J, Feldman M, Tong L, Shermer R (2003). The role of provider attitudes in prescribing antidepressants to older adults: leverage points for effective provider education. Int J Psychiatry Med.

[8] Chen S-Y, Hansen RA, Gaynes BN, Farley JF, Morrissey JP, Maciejewski ML (2010). Guideline-concordant antidepressant use among patients with major depressive disorder. Gen Hosp.

[9] Hepner KA, Rowe M, Rost K, Hickey SC, Sherbourne CD, Ford DE (2007). The effect of adherence to practice guidelines on depression outcomes. Ann Intern Med.

[10] Baker R, Camosso-Stefinovic J, Gillies C, Shaw EJ, Cheater F, Flottorp S (2015). Tailored interventions to address determinants of practice. The Cochrane Database Syst Rev..

[11] Aakhus E, Flottorp S, Vandvik PO, Brandt L, Oxman A. Guidelines for the management of depression in primary health care, and their relevance for the depressed elderly: a systematic review. Protocol. 2011. doi:10.15124/CRD42011001582. http://www.crd.york.ac.uk/PROSPERO/display_record.asp?ID=CRD42011001582.

[12] Unützer J (2007). Late-life depression. N Engl J Med.

[13] Alexopoulos GS (2005). Depression in the elderly. Lancet.

[14] Aakhus E, Oxman AD, Flottorp SA (2014). Determinants of adherence to recommendations for depressed elderly patients in primary care: a multi-methods study. Scand J Prim Health Care.

[15] Turner EH, Matthews AM, Linardatos E, Tell RA, Rosenthal R (2008). Selective publication of antidepressant trials and its influence on apparent efficacy. N Engl J Med.

[16] Hollon SD, Jarrett RB, Nierenberg AA, Thase ME, Trivedi M, Rush AJ (2005). Psychotherapy and medication in the treatment of adult and geriatric depression: which monotherapy or combined treatment?. J Clin Psychiatry.

[17] Pinquart M, Duberstein PR, Lyness JM (2006). Treatments for later-life depressive conditions: a meta-analytic comparison of pharmacotherapy and psychotherapy. Am J Psychiatry.

[18] Jager C, Freund T, Steinhauser J, Aakhus E, Flottorp S, Godycki-Cwirko M (2014). Tailored implementation for chronic diseases (TICD): a protocol for process evaluation in cluster randomized controlled trials in five European countries. Trials..

[19] Wensing M, Huntink E, van Lieshout J, Godycki-Cwirko M, Kowalczyk A, Jäger C (2014). Tailored implementation of evidence-based practice for patients with chronic diseases. PLoS One.

[20] Huntink E, van Lieshout J, Aakhus E, Baker R, Flottorp S, Godycki-Cwirko M (2014). Stakeholders contributions to tailored implementation programs: an observational study of group interview methods. Implement Sci..

[21] Flottorp SA, Oxman AD, Krause J, Musila NR, Wensing M, Godycki-Cwirko M (2013). A checklist for identifying determinants of practice: a systematic review and synthesis of frameworks and taxonomies of factors that prevent or enable improvements in healthcare professional practice. Implement Sci..

[22] Gurses AP, Murphy DJ, Martinez EA, Berenholtz SM, Pronovost PJ (2009). A practical tool to identify and eliminate barriers to compliance with evidence-based guidelines. Jt Comm J Qual Patient Saf..

[23] Osborn AF (1963). Applied imagination; principles and procedures of creative problem-solving.

[24] Glenton C, Colvin CJ, Carlsen B, Swartz A, Lewin S, Noyes J (2013). Barriers and facilitators to the implementation of lay health worker programmes to improve access to maternal and child health: qualitative evidence synthesis. Cochrane Database Syst Rev.

[25] Godycki-Cwirko M, Zakowska I, Kosiek K, Wensing M, Krawczyk J, Kowalczyk A (2014). Evaluation of a tailored implementation strategy to improve the management of patients with chronic obstructive pulmonary disease in primary care: a study protocol of a cluster randomized trial. Trials..

[26] Huntink E, Heijmans N, Wensing M, van Lieshout J (2013). Effectiveness of a tailored intervention to improve cardiovascular risk management in primary care: study protocol for a randomised controlled trial. Trials..

[27] Jager C, Freund T, Steinhauser J, Joos S, Wensing M, Szecsenyi J (2013). A tailored implementation intervention to implement recommendations addressing polypharmacy in multimorbid patients: study protocol of a cluster randomized controlled trial. Trials..

[28] Krause J, Agarwal S, Bodicoat D, Ring A, Shepherd D, Rogers S (2014). Evaluation of a tailored intervention to improve management of overweight and obesity in primary care: study protocol of a cluster randomised controlled trial. Trials..

[29] Piek E, Nolen WA, van der Meer K, Joling KJ, Kollen BJ, Penninx BW (2012). Determinants of (non-)recognition of depression by general practitioners: results of the Netherlands Study of Depression and Anxiety. J Affect Disord.

[30] McPherson S, Armstrong D (2012). General practitioner management of depression: a systematic review. Qual Health Res.

[31] Shirazi M, Lonka K, Parikh SV, Ristner G, Alaeddini F, Sadeghi M (2013). A tailored educational intervention improves doctor’s performance in managing depression: a randomized controlled trial. J Eval Clin Pract..

[32] Verhaak PF, Dekker JH, de Waal MW, van Marwijk HW, Comijs HC (2014). Depression, disability and somatic diseases among elderly. J Affect Disord.

[33] Baker R, Reddish S, Robertson N, Hearnshaw H, Jones B (2001). Randomised controlled trial of tailored strategies to implement guidelines for the management of patients with depression in general practice. Br J Gen Pract.

[34] Krause J, Van Lieshout J, Klomp R, Huntink E, Aakhus E, Flottorp S (2014). Identifying determinants of care for tailoring implementation in chronic diseases: an evaluation of different methods. Implement Sci..

[35] Wensing M, Huntink E, van Lieshout J, Godycki-Cwirko M, Kowalczyk A, Jager C (2014). Tailored implementation of evidence-based practice for patients with chronic diseases. PLoS One.

